# Molecular Authentication, Phytochemical Evaluation and Asexual Propagation of Wild-Growing *Rosa canina* L. (Rosaceae) Genotypes of Northern Greece for Sustainable Exploitation

**DOI:** 10.3390/plants10122634

**Published:** 2021-11-30

**Authors:** Eleni Maloupa, Eleftherios Karapatzak, Ioannis Ganopoulos, Antonis Karydas, Katerina Papanastasi, Dimitris Kyrkas, Paraskevi Yfanti, Nikos Nikisianis, Anthimos Zahariadis, Ioanna S. Kosma, Anastasia V. Badeka, Giorgos Patakioutas, Dimitrios Fotakis, Nikos Krigas

**Affiliations:** 1 Institute of Plant Breeding and Genetic Resources, Hellenic Agricultural Organization Dimitra, P.O. Box 60458, 57001 Thermi, Thessaloniki, Greece; maloupa@bbgk.gr (E.M.); ekarapatzak@gmail.com (E.K.); giannis.ganopoulos@gmail.com (I.G.); euripidis_1999@hotmail.com (A.K.); papanastasi@bbgk.gr (K.P.); 2 Department of Agriculture, University of Ioannina, 47100 Arta, Greece; dkyrkas@uoi.gr (D.K.); pyfanti@uoi.gr (P.Y.); gpatakiu@uoi.gr (G.P.); 3 Systada General Partnership, 55133 Kalamaria, Thessaloniki, Greece; nnikisia@gmail.com; 4 Verus plus General Partnership, 55133 Kalamaria, Thessaloniki, Greece; zachariadis@verusplus.com; 5 Laboratory of Food Chemistry, Department of Chemistry, University of Ioannina, 45110 Ioannina, Greece; i.kosma@uoi.gr (I.S.K.); abadeka@uoi.gr (A.V.B.); 6 Forest Research Institute, Hellenic Agricultural Organization Dimitra, 57006 Vassilika, Thessaloniki, Greece; fotakis@fri.gr

**Keywords:** biodiversity, ex-situ conservation, protocols, DNA barcoding, germplasm, phytogenetic resources, forest berries

## Abstract

Dogroses belong to a taxonomically difficult genus and family and represent important phytogenetic resources associated with high ornamental, pharmaceutical-cosmetic and nutritional values, thus suggesting a potentially high exploitation merit. Triggered by these prospects, wild-growing *Rosa canina* populations of Greece were selected for investigation and evaluation of their potential for integrated domestication. We collected ripe rosehips from Greek native wild-growing populations (samples from seven genotypes) for phytochemical analysis (total phenolics, total flavonoids, antioxidant activity and vitamin C content), leaf samples for DNA analysis using the ITS2 sequence (nine genotypes) and fresh soft-wood stem cuttings for propagation trials (seven genotypes). After evaluation of these materials, this study reports for the first-time distinct DNA-fingerprinted genotypes from Greece with interesting phytochemical profiles mainly in terms of Vitamic C content (up to 500.22 ± 0.15 mg of ascorbic acid equivalents/100 g of sample) as well as effective asexual propagation protocols for prioritized *R. canina* genotypes via cuttings. The latter highlights the importance of the levels of external hormone application (2000 ppm of indole-3-butyric acid), the effect of season (highly-effective spring trials) and genotype-specific differences in rooting capacities of the studied genotypes. All inclusive, this study offers new artificially selected material of Greek native *R. canina* with a consolidated identity and interesting phytochemical profile. These materials are currently under ex-situ conservation for further evaluation and characterization in pilot field studies, thus facilitating its sustainable exploitation for applications in the agro-alimentary, medicinal-cosmetic, and ornamental sectors.

## 1. Introduction

To date there are at least 373 recognized species worldwide in genus *Rosa* L. of Rosaceae family (www.theplantlist.org, accessed on 1 November 2021) and about 30,000 ornamental varieties [1,2]; the latter are probably derived from only seven species largely contributing to the creation of the modern commercial rose with further seven species providing some minor inputs [3]. Nevertheless, according to the Plant List database 19 rose species are still unplaced due to unresolved taxonomic status, and additionally >3100 species names of roses remain to be evaluated, thus outlining a notoriously complex taxonomy in genus *Rosa* [4]. Regarding the European dogroses of section *Caninae* (DC.) Ser., two basic trends currently prevail; they are either well-regarded by rose lovers for their attractiveness and pleasant scent or they are dreaded by scientists for their genetic complexity [5]. With about 60 rose species of Eurasian distribution, *Caninae* represents one of the largest sections of the genus *Rosa* [5]. The species included in this section cannot be well circumscribed by specific morphological traits due to multiple reproductive strategies (from apomixis to outcrossing including hybridization) and a unique meiotic system (the so-called canina meiosis), rendering dogroses as mostly pentaploids (rarely tetraploids or hexaploids; 2 n = 4 x, 5 x, 6 x = 28, 35, 42) with a base number of seven chromosomes [5]. The complex taxonomy of this family, genus, and section makes species identification difficult, and genetic studies including DNA barcoding may offer insight regarding relationships among closely related species [6]. 

The systematic genetic identification of model and non-model organisms through the use of modern molecular techniques based on the DNA sequence has become very popular in recent years. The DNA barcoding method is a widely used molecular tool which in combination with bioinformatic analysis has a wealth of applications in ecological, taxonomical, comparative biology, diversity, conservation, phylogenic and genetic studies amongst various plant species and taxonomic groups [7], but also in members of the Rosaceae family [6] and of the genus *Rosa* (e.g., [2,8,9,10,11]). Different DNA fingerprinting techniques were applied in members of the genus *Rosa* for effective characterization [12,13] using different molecular markers such as Inter Simple Sequence Repeats (ISSRs) [2,10], Random Amplified Polymorphic DNA (RAPD) markers [8], Internal Transcribed Spacer (ITS) markers [6] and Amplified Fragment Length Polymorphisms (AFLPs) markers [14]. From the plethora of markers that have been evaluated to date for barcoding (mainly of chloroplastic DNA, e.g., rbcL, matK, trnH-psbA, etc.), the nuclear internal transcribed spacer 2 (ITS2) is the predominant one due to its short length, high efficiency in species differentiation and clear PCR amplification results [15]. Studies including thousands of samples from 4800 medicinal plant species in 753 distinct genera [15] and of 893 Rosaceae members in 96 genera [6] suggest that ITS2 is the most suitable and effective DNA barcoding marker for identification purposes both in general and specifically in Rosaceae members including members of genus *Rosa*. 

The first sequencing of the rose genome has been performed only recently [16] by sequencing of wild and heterozygous *Rosa multiflora* Thunb. genotypes. Two high quality reference genomes have been published which significantly enrich existing information, i.e., a high-density diploid SNP genetic map [17] and a high-density map for tetraploid rose crucial for anchoring pseudomolecules corresponding to the chromosomes [18]. These results were validated by HiC sequencing [19], reporting genes and transposable element annotation. During the last decade, next-generation sequencing has released transcriptome data from numerous tissues and developmental stages for various genotypes and rose species [20,21,22]. All these data accumulated to date are of paramount importance for gene annotation studies and such data and metadata may pave the way for the construction of a gene expression atlas anchoring candidate genes of interest through meta-analyses in several members of the genus *Rosa*. 

Rose hips of several *Rosa* spp. of section *Caninae* are used in many regions worldwide (also in the Mediterranean region) for tea, soup, jam and jelly preparations [23,24]. Rose hips contain major biologically active components such as flavonoids, tannins, anthocyanins, phenolic compounds, fatty oils, organic acids as well as inorganic compounds [24]. Among dogroses, *R. canina* L. produces fruits (rose hips) of high pharmaceutical [24,25,26] and nutritional value [24]. *Rosa* spp. have been reported to be rich in several biologically active compounds such as high vitamin C content exceeding that of Citrus fruits. Other active compounds of *Rosa* spp. among others are phenolics, flavonoids, tannins, organic acids, etc. [27,28]. Due to their rich content in bioactive molecules associated with beneficial properties and few toxic or allergic reports and side effects [24], rose hips (including those of *R. canina*) are used in many countries for the treatment of many symptoms such as pain, gastroenteric ailments, cough, cold, inflammations, diarrhea etc., as well as for the prevention of many diseases including diabetes, hypertension, bronchitis, flu, arthritis etc. [24,28,29,30,31]. Previous targeted studies have shown that rose hips (including those of *R. canina*) demonstrate significant antioxidant, anticancer, anti-inflammatory, anti-obesity, anti-aging, antinociceptive, anti-Helicobacter pylori activities as well as gastro-, hepato-, nephro-, neuro- and cardioprotective activities [24,28,29,30,31]. Although *R. canina* is well-studied plant species in terms of phytochemisty, traditional uses and pharmacological profile [24], still there are very few studies examining wild-growing material from Greece, e.g., [28].

In the European context, Greece is quite rich in different *Rosa* spp. (http://portal.cybertaxonomy.org/flora-greece/intro accessed on 1 November 2021), including native wild-growing populations of 23 distinct species, thus almost half of the European *Rosa* species listed in Flora Europaea [32]. *R. canina* is widespread in Greece across a variety of habitat types (slopes, valleys, riverbanks, usually in thickets and open woodland along roads) from sea level up to 1600–1700 m (occasionally up to 2000 m). It flowers mainly in June and ripe fruits in wild-growing populations are usually observed from the end of August to the beginning of October [33]. For the successful utilization and exploitation of Greek native *R. canina* germplasm, the development of a distinct and solid identity based on DNA fingerprinting is aimed in the first place, elucidating relationships with other relevant materials. Furthermore, the development of effective asexual propagation protocol is crucial, safeguarding the steady transfer of desirable agronomic features and fruit traits to the offspring on one hand, and securing the production of uniform plant material on a commercial scale on the other hand. The use of cuttings is a very efficient and cost-efficient asexual propagation method that can serve these needs [34]. Although the propagation via cuttings of *R. canina* has been conducted on a variety of germplasm sources highlighting the enhancing effect on rooting of external application of indole-3-butyric acid (IBA) [35,36,37], there are no such studies however targeted to Greek native germplasm of *R. canina*. 

In the frame of sustainable exploitation strategies [38,39,40] and coordinated research efforts to explore and evaluate the economic potential of neglected and underutilized phytogenetic resources which are native to Mediterranean regions [41,42,43], the aim of the current study was three-fold, focusing on: (i) the molecular authentication of Greek native *R. canina* germplasm (DNA fingerprinting of different genotypes); the evaluation of phytochemical content of selected Greek native genotypes; and (iii) the development of genotype-specific asexual propagation protocols thereof by cuttings. The study was conducted in two phases: Firstly, the implementation of preliminary propagation trials was performed from material collected directly from the wild using a variety of hormone levels and cutting types across different periods of the year, namely different growth stages of mother plants. This was done in order to be able to assess roughly the propagation potential of different *R. canina* genotypes. In parallel, wild-collected leaves were used for DNA barcoding, and wild harvested rose hips were used for phytochemical assessments of different genotypes. Consecutively, following-up the preliminary propagation results, and taking into account the overall assessment of molecular authentication and general phytochemical assessments of the studied Greek native genotypes, targeted propagation experiments were conducted on selected materials using ex-situ raised mother plants which originated in the wild. The consolidated identity of genotypes, the phytochemical profile and the development of a reliable, easy to implement and economically viable propagation protocols are aimed herein to further contribute to the sustainable utilization of the selected Greek native germplasm of *R. canina*.

## 2. Results

### 2.1. Authentication Efficiency of ITS2

**Figure 1:**
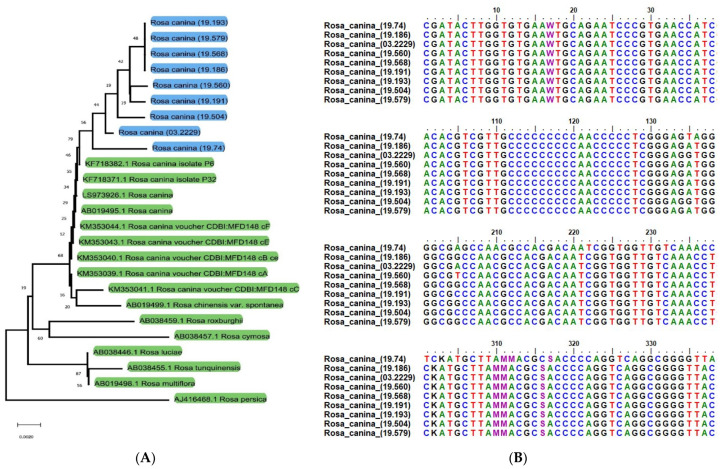
Phylogenetic tree (**Α**) constructed on the basis of ITS2 regions of the Greek native *Rosa canina* genotypes contrasted with other *R. canina* and *Rosa* spp. genotypes retrieved from NCBI with multiple sequence alignment of the ITS2 bar-code region of the genotypes analyzed in this study (**B**). Results from neighbor-joining (NJ) bootstrap analyses with 500 replicates was used to assess the strength of the nodes. The node numbers indicated the bootstrap value of NJ. The distinct genotypes of this study are highlighted with blue.

To test the authentication efficiency of ITS2 for the selected Greek native *Rosa canina* genotypes, the BLAST1 and distance-based methods were selected. Using the BLAST1 method, the ITS2 barcode showed a high identification efficiency of 99% and 100% of the samples at the species and genus levels, respectively. Additionally, using the DISTANCE method, the ITS2 barcode showed identification efficiency of 98% at the species level. Figure 1A depicted that barcode ITS2 using the NJ tree method is able to distinguish Greek native *Rosa canina* genotypes from *R. canina* genotypes which are not native to Greece as well as from other *Rosa* spp. The neighbor-joining (NJ) phylogenetic tree resulting from DNA barcoding application using the ITS2 region classified all *Rosa* spp. samples into four different groups, and clearly discerned the Greek native *R. canina* samples collected in this study from all other samples of *R. canina* sourced from databases (Figure 1A). Bootstrap values further validate this classification. Although evolutionary relationships may be analyzed through the neighbor-joining tree, its key function herein is to repetitively evaluate bootstrap values to enlighten distinction of the Greek native germplasm (see distinct clades). Our results showed that the ITS2 gene is a valid choice with absolute (100%) efficiency for the distinction of the studied species (*R. canina*) among *Rosa* spp., classifying each *Rosa* species in separate monophyletic clusters.

The sequence of each amplicon and the alignment between *R. canina* Greek native genotypes is depicted in Figure 1B. The sequences of the *R. canina* genotypes studied herein possessed variation capable to produce differences in phylogenetic analysis. Specifically, we observed a number of single nucleotide polymorphisms (SNPs) which is responsible for the detected differences in the phylogenetic analysis. The evolutionary distances were computed using the Maximum Composite Likelihood method [44] and are presented in the units of the number of base substitutions per site. This analysis involved 25 nucleotide sequences. Codon positions included were 1st + 2nd + 3rd + Noncoding. All ambiguous positions were removed for each sequence pair (pairwise deletion option). There were 391 positions in total in the final dataset discriminating and classifying the Greek native *R. canina* genotypes from other genetically related genotypes or species. The constructed NJ tree of phylogenetic relationships included the Greek accessions of *R. canina* as a separate branch with 60% branch support. Therefore, with the use of the nuclear ITS2 barcoding sequence, the Greek native *R. canina* genotypes could be sufficiently distinguished from others which are not native to Greece, and from other closely related species genetically. Thus, using the ITS2 barcoding region, the nine *R. canina* genotypes studied herein were fingerprinted.

### 2.2. Phytochemical Analysis of Greek Native Rosa canina Rosehips

Table 1 shows the results as mean values and standard deviations (SD) of the total phenolic content (TPC), antioxidant activity (AA), total flavonoid (TF) and Vitamin C content of the Greek native *R. canina* samples analyzed (wild-growing plant material). Regarding TPC values, the lowest and highest values were recorded from GR-1-BBGK-O3,2229 (62.98 ± 0.01 mg GAE/100 g) and GR-1-BBGK-19,504 (215.46 ± 0.00 mg GAE/100 g), respectively, whereas statistically significant differences (*p* < 0.05) were found in most of the samples. On the other hand, AA showed limited variations as most samples presented AA values of the same order of magnitude, with the exception of GR-1-BBGK-19,568 which recorded the lowest AA (88.41% ± 0.46). Large variations with statistically significant differences (*p* < 0.05) were recorded in TF values; GR-1-BBGK-O3,2229 showed the lowest TF value (0.87 ± 0.01 mg CE/100 g) and GR-1-BBGK-19,674 the highest (2.46 ± 0.02 mg CE/100 g). Finally, Vitamin C content varied significantly (*p* < 0.05) among all studied samples. Most of the samples analyzed showed high content of Vitamin C as AAE/100 g as expected, with the exception of GR-1-BBGK-19,504 which showed the lowest value 71.85 ± 0.28 in relation to the other samples tested. 

### 2.3. Preliminary Propagation Trials

A broad spectrum of rooting capacity was observed between the studied *R. canina* genotypes following external hormone application in preliminary trials (Table 2). Differences in rooting capacity were observed among genotypes at different seasons, in different vegetative stages and using different cutting types (Table 2). In particular, the genotype GR-1-BBGK-19,191 showed 44% rooting of hardwood cuttings in winter when treated with 10,000 ppm IBA, whereas softwood leafy cuttings at early growth in the following spring treated with 2500 ppm IBA powder presented 75.7% rooting.

### 2.4. Assessment of Greek Native Rosa canina Genotypes

**Figure 2:**
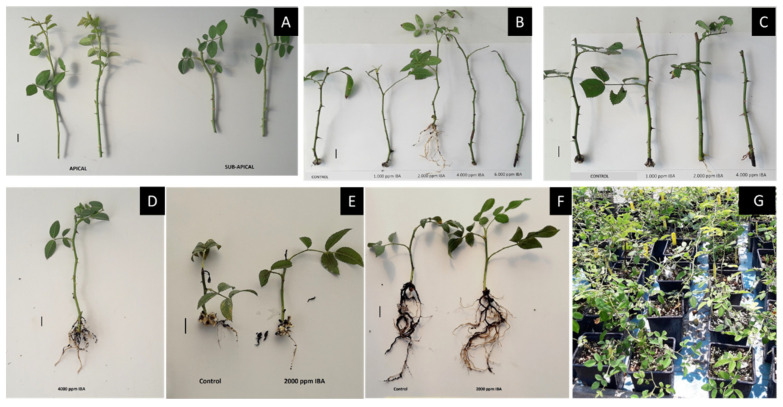
Representative photos of cutting propagation results (experiments in 2019 and 2020) on selected Greek native genotypes of *Rosa canina* originating form wild-growing material. (**A**) Softwood leafy cuttings (apical and sub-apical) of the prioritized genotype GR-1-BBGK-19,191; (**B**,**C**) Rooting results of apical and subapical cuttings of GR-1-BBGK-19,191, respectively, in 3:1 substrate across different hormone treatments tested; (**D**) Rooted cutting of the 4000 ppm IBA treatment in the 2020 experiment with the prioritized genotype GR-1-BBGK-03,2229; (**E**,**F**) Rooted cuttings of the 2020 experiment of the genotype GR-1-BBGK-19,674 (control and 2000 ppm IBA treatments) coming from mother plants with no fertilization (**E**) contrasted to those coming from mother plants with conventional fertilization in (**F**); (**G**) New plants of the genotype GR-1-BBGK-19,191 raised ex-situ under outdoor adaptation. Bars in photos A to F represent 1 cm.

Based hierarchically on: (i) the results of the molecular authentication achieved for nine Greek native *R. canina* genotypes (Figure 1 and Figure 2), (ii) the success assessments regarding the preliminary trials on seven genotypes (Table 2) and (iii) the relative phytochemical interest in terms of comparative vitamin C content and total phenolic content detected in seven genotypes (Table 1), the genotypes GR-1-BBGK-19,191 and GR-1-BBGK-03,2229 were prioritized as most promising for sustainable exploitation strategies. The prioritization was based on the fulfillment of three criteria, i.e., effective DNA barcoding, high success of propagation trials as well as strong phytochemical interest (comparatively very high or high vitamin C content and high or low total phenolic content). Table 3 characterizes the results obtained from the molecular analysis as effective due to the fact that Greek genotypes were grouped independently from others when genetically compared; outlines the success of propagation trials in terms of rooting percentage as low (<40%) or high (>40%); and provides insight regarding the most important phytochemical interest in terms of comparative vitamin C content (very high: >400; high: >340–400; Low: <340 mg /100 g) and total phenolic content (very high: >90; high: 80–90; low: <80 mg of gallic acid/100 g).

### 2.5. Experimentation on Asexual Propagation of R. canina 

Following the preliminary propagation results obtained (Table 1), when softwood cuttings of the more advanced growth of genotype GR-1-BBGK-19,191 were set again in a broader experiment during the summer, the rooting capacity reached 37.5% after 32 days under 2000–4000 ppm IBA (Table 4, Figure 2). However, this rooting capacity was reached through subapical cuttings treated with 2000 ppm IBA in 1:3 *v/v* peat/perlite and through apical cuttings treated with 4000 ppm IBA in 1:1 *v/v* peat/perlite (Table 4, Figure 2). Root number and root length on the other hand did not show to be significantly affected by hormone, cutting type or substrate (Table 2, *p* < 0.05). Similarly, in the genotype GR-1-BBGK-03,2229 which was studied across two consecutive years, rooting capacity took 42 days to reach 25% with soft wood cuttings of early growth during spring in 2019 both with 4000 ppm IBA in 1:1 *v/v* peat/perlite and 2500 ppm IBA powder in 1:3 *v/v* peat/perlite (Table 5, Figure 2). The same genotype during the following year, reached 66.7% rooting in 30 days with softwood cuttings taken when in advanced growth during the summer and treated with 4000 ppm IBA in 1:3 *v/v* peat/perlite, however without a statistically significant effect on root number or length (Table 5, *p* < 0.05). 

In another experiment during the summer of 2020 with genotype GR-1-BBGK-19,674 and mother plants grown in a cultivation trial under different fertilization regimes, rooting capacity of softwood cuttings coming from donor plants treated with conventional fertilization reached 50% under 2000 ppm IBA (Table 6). 

## 3. Discussion

### 3.1. Molecular Authentication of Greek Native Genotypes of Rosa canina

DNA barcoding is a valid technique for the discrimination of *R. canina* genotypes since it is not affected by the stage of plant development and may further enhance the classical morphological identification offering insight regarding phylogenetic relationships of closely related species. In this study, the first-ever report regarding the molecular authentication of Greek native germplasm of *R. canina* is provided. The NJ (Neighbor-Joining) tree classification resulting from the use of barcoding technique in conjunction with ITS2 gene was in accordance with internationally accepted phylogenetic relations, and allowed the distinction of specific genotypes within the species *R. canina*. Using the ITS2 barcoding region, the nine Greek native *R. canina* genotypes studied herein were fingerprinted, and they were clearly separated from other genotypes of *R. canina* which are not native to Greece or other members in the genus Rosa. However, to further confirm the application of this barcoding technique using the ITS2 sequence, different species of genus *Rosa* from different habitats in different regions of Greece should be evaluated. Thus, ITS2 gene can be an effective and valid marker for the identification of the species and of different genotypes of *R. canina*, enriching the extant knowledge regarding the elucidation of evolutionary relationships and classification of Rosaceae and *Rosa* members.

### 3.2. Phytochemical Potential of Greek Native Genotypes of Rosa canina

The study herein represents the first comprehensive report of TPC, TF, AA and Vitamin C content of rosehips from Greek native genotypes of *Rosa canina*. The TPC values of the samples analyzed showed lower values compared to that reported previously in a study examining a single sample of Greek native plant material [28]; only GR-1-BBGK-19,504 showed TPC value in the same order of magnitude (215.46 ± 0.00 mg GAE/100 g) and this was the highest value detected among the studied Greek native genotypes. Previous studies [29] testing eight samples of *R. canina* from Transylvania, Romania have reported higher values than those detected in the present study, from 326.3 ± 5.65 to 575.1 ± 14.64 mg GAE/100 g of frozen rose hip pulp. 

Regarding the TF content, [27] report average TF content of *R. canina* samples from Azerbaijan 2.02 ± 0.03 mg quercetin /100 g, which is slightly higher than that detected in samples of the present study (1.80 ± 0.50 mg CE/100 g). Among the studied Greek genotypes herein, GR-1-BBGK-19 showed above-average potential in terms of TF content and ranked comparatively high. 

AA values reported previously in samples of *R. canina* from Azerbaijan [27] are lower than those detected herein for the Greek native samples (94.53% ± 2.59 vs. 83.41% ± 0.86). Among the studied Greek genotypes herein, GR-1-BBGK-19,191, GR-1-BBGK-03,2229 and GR-1-BBGK-19,504 were ranked comparatively above-average in terms of TF content. 

Finally, Vitamin C content of the *R. canina* samples studied herein presented higher values (average 354.50 ± 128.21 mg AAE/100 g) compared to other investigations, e.g., from 112.20 ± 2.82 mg AA/100 g to 360.22 ± 2.87 mg AA/100 g [29]. Among the Greek genotypes of *R. canina* studied herein, the higher content was found in GR-1-BBGK-19,568 (500.22 ± 0.15 mg AA/100 g) followed by GR-1-BBGK-19,191.

### 3.3. Propagation Potential of Greek Native Genotypes 

In the current study, rooting data of cutting propagation for Greek native *R. canina* germplasm are presented for the first time. From a commercial perspective, the rooting capacities observed herein are similar to those observed for *R. canina* genotypes in other studies [35,36,45] and can be considered above the threshold for commercial efficacy. With adequate mother plant growth, rooting capacity above 50% (namely for every two cuttings obtained from mother plants at least one successfully delivers a new plant) can be considered as commercially acceptable [34]. 

The Greek native *R. canina* (dogrose) genotypes studied herein have shown diversified propagation potential in terms of rooting capacity of cuttings. Different genotypes presented differences in propagation performance under different rooting factors, which suggests an interaction between genotype and the external factors involved in the rooting of cuttings. In previous propagation studies of *R. canina* genotypes with the use of IBA, a significant interaction between time of year and genotype was suggested [36], in the same fashion to the current results. Similarly, it has been suggested an effect of developmental stage of donor plants on hormone translocation and uptake by cuttings [36]. A significant effect of genotype on rooting of hardwood cuttings of *R. canina* and other rose hips has also been observed in earlier studies with indigenous eastern Mediterranean germplasm [46], including the promoting effect that IBA has on the rooting of cuttings [35]. In the current study, different types of cuttings of the same genotype showed varied rooting capacity at different annual growth stages of the donor material, consistently, however, requiring external hormone application. This observation is in agreement with similar results on the effects of donor plants’ growth stage, cutting type and IBA on the rooting of cuttings in commercial germplasm of *R. hybrida* E. H. L. Krause grown in Mediterranean conditions [47]. Additionally, notable differences in cuttings’ response to hormonal regimes between genotypes have also been observed in *R.* x *damascena* Herrm. [45]. Given that the cuttings conditions set for rooting were similar between different trials, this trend suggests that the effect of season is probably related to the growth developmental stage during which cuttings are taken. The different cutting types seem to indicate a genotype-varying response to the external hormone application. The effect of season on rooting of *Rosa* cuttings has been suggested as a significant factor by other investigators stemming from results of similar studies [48,49]. 

The effect of cutting type has been attributed by other studies to the differences in the nutrient status and carbohydrate concentration of the cuttings coming from different parts of the mother plant, as reported in *R. hybrida* commercial rootstock germplasm [50]. However, a clear conclusion cannot be drawn on the particular effect of cutting type in the current study since the nutritional status or carbohydrate balance of the Greek native *R. canina* germplasm studied herein have not yet been adequately studied and further research is needed. In addition, other studies dealing with rooting patterns across genotypes of native germplasm of *R. canina* suggest that the treatment of mycorrhizal fungi as well as growth promoting bacteria have possible synergistic effects on the rooting of cuttings [51,52]. Furthermore, grafting should be also examined as a method to overcome genetic differences at ease of propagation; this consists of grafting a desirable, difficult to root, genotype onto another genotype that is stronger in terms of rooting capacity. It is known that grafting is widely being performed in commercial roses [53]. However, there is evidence that grafting can affect plant physiology of interacting genotypes [54,55]. Consequently, caution should be taken when desirable fruit characteristics of the scion are involved such as natural content of vitamin C or total phenolics content. Undoubtedly, further research and experimentation is suggested regarding the potential implementation of grafting on Greek native *R. canina* germplasm. 

## 4. Materials and Methods 

### 4.1. Plant Populations of Rosa canina Sampled

**Figure 3:**
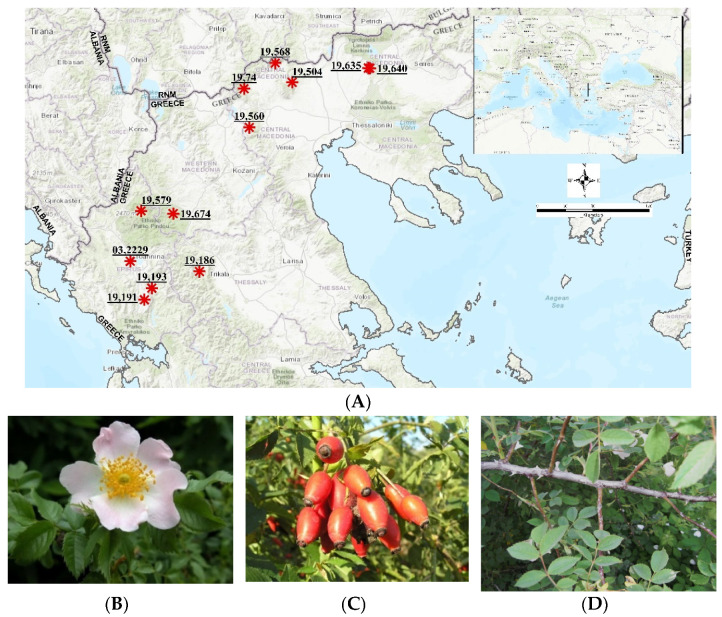
Overview of the collection sites of the *Rosa canina* Greek native germplasm analyzed (**A**), and morphology of flowers (**B**), fruits (**C**), and leaves (**D**) of *R. canina* GR-1-BBGK-03,2229 used for taxonomic identification, phytochemical analysis and DNA barcoding, respectively (for IPEN accession numbers see Table 7).

Nine authorized botanical expeditions were organized in 2019 to explore different areas of Northern Greece (Epirus and North-central Greece) for wild-growing *R. canina* initial materials with vigorous growth and strong fruiting potential in the wild habitats. The collections were performed using the authorized special permit of the Institute of Plant Breeding and Phytogenetic Resources, Hellenic Agricultural Organization Demeter (Permit 82336/879 of 18/5/2019 & 26895/1527 of 21/4/2021). This permit is issued yearly by the Greek Ministry of Environment and Energy after detailed reporting of the applicant. The collections were performed in the frame of the research project “Highlighting local traditional varieties and wild native forest fruit trees and shrubs” (acronym: EcoVariety, Τ1ΕΔΚ-05434). In each expedition (Table 7, Figure 3, we collected from selected wild-growing *R. canina* populations (Greek native germplasm): (a) sets of fresh soft-wood stem cuttings as initial propagation material for propagation trials (in total, seven populations), (b) ripe rosehips sampled from three individuals for phytochemical analysis (in total, seven populations), and (c) leaf samples from 20 individuals destined for DNA analysis (in total, nine populations). The materials were taxonomically identified based on standard diagnostic keys for the European [32] and Greek *Rosa* material [33]. Consequently, each genotype was given a unique IPEN (International Plant Exchange Network) accession number by the Institute of Plant Breeding and Genetic Resources (IPB&GR) of the Hellenic Agricultural Organization Demeter. 

### 4.2. DNA Isolation 

Approximately 30 mg of dried leaf sample was completely grounded in liquid nitrogen. Total DNA was isolated from leaf samples of *R. canina* using a Nucleospin Plant II (Macherey-Nagel) kit following the manufacturer’s instructions.

### 4.3. Polymerase Chain Reaction (PCR) Amplification

One primer set of the nuclear ITS2 barcode region suggested by [56] was used for amplification and sequencing. The PCR amplification was performed according to [57]. 

### 4.4. Sequence Analysis 

PCR products were directly sequenced in two directions of each fragment with a Big Dye terminator v3.1 Cycle sequencing kit (PE Applied Biosystems, Foster City, CA, USA) in an automated ABI 3730 sequencer (PE Applied Biosystems). The sequences were aligned using the CLUSTAL W program. 

### 4.5. Molecular Data Analysis

Three methods were employed for molecular authentication of the selected *R. canina* genotypes: (1) Basic Local Alignment Search Tool (BLAST) search using the nucleotide database at NCBI [58]; (2) the genetic divergence method using maximum-likelihood models; and (3) tree topology analysis based on the neighbor-joining (NJ) method based on different loci in MEGAX [59] with the K2P distance model and 500 bootstrap replications. The sequences obtained after removing the primers used for PCR amplification were deposited to NCBI-Genbank BankIt (https://www.ncbi.nlm.nih.gov/BankIt/, accessed on 1 November 2021) under the accession numbers MK5334116 to MK5334124.

### 4.6. Phylogenetic Relationships 

The phylogenetic relationships of different *Rosa* spp. were inferred using the Neighbor-Joining method [60]. The optimal tree with the sum of branch length = 0.07053901 is shown. The percentage of replicate trees in which the associated taxa clustered together in the bootstrap test (500 replicates) are shown next to the branches [61]. Phylogenetic analyses were conducted in MEGA X [59].

### 4.7. Phytochemical Analysis of Rosa canina Rosehips 

The extracts were prepared according to the method described by [62] with some modifications. Part of the homogenized sample (2–5 g) was mixed with an appropriate amount of MeOH/H_2_O (60:40), and the mixture was centrifuged (4 °C, 4000 rpm). The supernatant was collected and the volume was made up to 20 mL. This extract was used for the following analyses:

Total phenolic content (TPC): The determination of TPC was carried out using the method described by [62]. Phenolic extract 0.20 mL along with 2.3 mL of H_2_O and 0.25 mL of Folin-Ciocalteu reagent were added in a volumetric flask. After 3 min, 0.50 mL of 20% Na_2_CO_3_ was added, and the volume was made up to 5 mL. The solution was stored in a dark place for 2 h. After 2 h the absorbance was measured at 725 nm against blank solution. Total phenolics were calculated using a standard curve of gallic acid at various concentrations. The results given are expressed as gallic acid equivalents (GAE)/100 g of sample. All analyses were carried out in triplicate. 

Total Flavonoids (TF): The determination of TF was carried out according to the method of [29], with some modifications. Firstly, 5 μL of the above extract along with 3270 μL of H_2_O and 75 μL of 5% NaNO_2_ were added to a test tube, stirred, and stored in the dark for 5 min. After that, 150 μL of 10% AlCl_3_-6H_2_O was added, mixed, and stored again in the dark for 6 min. Then, 500 μL of 1 M NaOH was then added, and the absorbance was measured at 510 nm against H_2_O as blank. Total flavonoids were calculated using a standard curve of catechin at various concentrations. The results given are expressed as catechin equivalents (CE)/100 g of sample. All analyses were carried out in triplicate. 

Antioxidant Activity (AA): The determination of AA was carried out according to the method described by [27], with some modifications. Phenolic extract 0.1 mL along with 2.9 mL 0.10 mM DPPH in MeOH were added in a 5 mL plastic cuvette, stored in a dark place for 15 min and then the absorbance was measured at 517 nm against MeOH as blank. The control sample was prepared daily using only 0.10 mM DPPH in MeOH. The percentage of radical scavenging activity (%RSA) was calculated using the following equation:

%RSA = (Ao − As)/Ao × 100,

where

Ao = Absorbance of control sample

As = Absorbance of the sample after 15 min of incubation

All analyses were carried out in triplicate. 

Determination of Vitamin C: The determination of Vitamin C was carried out according to the method described by [63] after some modifications. A certain amount of homogenized sample (2–5 g) was added to a centrifuge tube with 5 mL of 4.5% *w/v* metaphosphoric acid (MPA) solution. The mixture was stirred and centrifuged at 8000 rpm at 4 °C for 20 min. Then, 1 mL of the supernatant was taken and diluted up to 10 mL with 4.5% MPA solution. This solution was filtered through 0.45 μm polyethersulfone filters. The vial was covered with aluminium foil to prevent oxidation of ascorbic acid and stored at 4 °C until HPLC-DAD analysis. HPLC-DAD conditions: Column (Agilent Eclipse XDB-C18) 4.6 mm × 150 mm, 5μm, elution solvent: aqueous 0.005 M H_2_SO_4_ solution at a flow rate of 0.5 mL/min (isocratic) and wavelength 245 nm. Vitamin C was calculated using a standard curve of ascorbic acid at various concentrations. The results given are expressed as ascorbic acid equivalents (AAE)/100 g of sample. All analyses were carried out in triplicate. 

### 4.8. Preliminary Propagation Trials and Mother Plants’ Growth Conditions

The soft-wood stem cuttings of the seven *R. canina* genotypes sampled (Table 7) were tested for rooting under various external hormone application treatments of indole-3-butyric acid (IBA) in propagation trays with peat: perlite at 1:3 *v/v* (Table 1); during experimentation, they were maintained at ambient temperature on mist bench in a plastic greenhouse with relative humidity (RH) maintained >85% where they were attended weekly to assess their rooting capacity. The produced mother plants were kept ex-situ at the grounds of IPB&GR under ambient conditions. The plants were watered regularly and were grown in 3 L pots using a mixture of peat and perlite (1:3 *v/v*). This allowed vigorous growth of mother plants, which enabled the raising of enough plant material for further experimentation during the next season.

### 4.9. Propagation Experimental Design, Cutting Types, Hormone Applications and Rooting Conditions

Following the preliminary observations (see Table 2 in results), two experiments were conducted in 2019 and another two experiments in 2020 regarding prioritized *R. canina* genotypes. In particular, an experiment was conducted on genotype GR-1-BBGK-19,191 in summer of 2019 which abided by a complete randomised design with five hormone application levels of Indole-3-butyric acid (IBA) (control; 1000 ppm; 2000 ppm; 4000 ppm; and 6000 ppm dissolved in 50% ethanol), two cutting types (1st year softwood apical and sub-apical cuttings) and two substrates (peat: perlite at 1:3 *v/v* and at 1:1 *v/v*), resulting in 20 treatments in total with eight replicate cuttings per treatment. Three fully developed leaves were kept in all cuttings. 

Another experiment was conducted on genotype GR-1-BBGK-03,2229 in May 2019. This experiment was set in a complete randomized block design with two blocks each with five IBA application levels (control; 1000 ppm; 2000 ppm; 4000 ppm dissolved in 50% ethanol and 0.25% powder) and two substrates (peat: perlite at 1:3 *v/v* and at 1:1 *v/v*), resulting in 10 treatments in total with eight replicate cuttings per treatment in each block. Following the results of 2019 regarding GR-1-BBGK-03,2229, another experiment was conducted in July 2020 in a complete randomized block design with two blocks each consisted of three IBA levels (control; 2000 ppm; and 4000 ppm dissolved in 50% ethanol), resulting in three treatments with six replicate cuttings per treatment in each block. The substrate used was peat: perlite at 1:3 *v/v*. Cuttings in both years were of the same type (soft-wood leafy cuttings).

In addition, during 2020, further experimentation was performed on genotype GR-1-BBGK-19,674 using field established mother plants following the project’s progress and taking into account the preliminary results. This experiment was conducted in a split-plot design having the fertilization status of mother plants in three main plots (no fertilization; conventional fertilization; organic fertilization) each having two sub-plots of IBA treatment (control and 2000 ppm dissolved in 50% ethanol). The six resulted treatments/sub-plots had six replicate cuttings each. The substrate used for rooting was peat: perlite at 1:3 *v/v*.

### 4.10. Cuttings’ Performance and Growth Measurements 

Observations on the progress of cuttings were taken weekly. When a treatment reached 100% rooting or after 40 days (whatever occurred first), the trays were taken out of mist and measurements were taken on rooting capacity, root number and root length per cutting. At the same time, rooted cuttings were transplanted in 0.5 L pots with peat: perlite 3:1 *v/v* substrate and were kept for the first two weeks within a greenhouse with shading and automated irrigation for plant establishment. 

### 4.11. Statistical Analysis of Rooting Data and Phytochemical Data

The rooting data were subjected to analysis of variance (GLM-ANOVA) to establish overall treatment effects and the phytochemical data were analysed using one-way ANOVA. Consecutively, and following the results of the ANOVA, to dissect specific treatment effects (hormone level, cutting type, substrate type) on the variables measured, data were split and separate analyses of variance were conducted as appropriate. Means from rooting experiments and means of phytochemical data were compared separately using Tukey’s HSD post hoc test Rooting frequencies were compared through pairwise Pearson Chi-Square tests. All analyses were conducted using the IBM-SPSS 23.0 software.

## 5. Conclusions

In the frame of sustainable exploitation strategies involving neglected and underutilized phytogenetic resources and domestication of wild-growing genotypes of native plants, the study herein focused on the exploration of the potential of Greek native *Rosa canina* germplasm. This study reported for the first time data regarding: (i) The effectiveness of fingerprinting distinct genotypes from Greece using the ITS2 sequence as molecular marker; (ii) The diverse phytochemical content in terms of total phenolics, total flavonoids, antioxidant activity and vitamin C content of different genotypes naturally occurring in northern Greece with interesting potential for applications; (iii) The effective propagation of selected and prioritized *R. canina* genotypes via cuttings, highlighting at the same time the importance of levels of external hormone application (IBA), the effect of season in terms of annual growth stage of donor plants, and genotype-specific differences in rooting capacities. The multifaceted documentation developed and assessed in this study offer new artificially selected plant material with consolidated identity and interesting phytochemical profile which is currently under ex-situ conservation for further evaluation and characterization in pilot field studies. In this way, the present work may pave the way for the sustainable exploitation of the selected Greek native genotypes of *R. canina*, facilitating future applications in the agro-alimentary, medicinal-cosmetic and ornamental sectors.

## Figures and Tables

**Table 1:** Values of total phenolic content (TPC), antioxidant activity (AA), total flavonoids (TF) and Vitamin C content detected in samples of wild-growing genotypes of *Rosa canina* of northern Greece.

Population Sample	TPC (mg GAE/100 g)	AA (%RSA)	TF (mg CE/100 g)	Vitamin C (mg AAE/100 g)
GR-1-BBGK-19,191	83.56 ± 0.20 d	95.82 ± 0.50 a	1.44 ± 0.20 c	426.12 ± 0.52 b
GR-1-BBGK-03,2229	62.98 ± 0.01 f	95.36 ± 0.40 a	0.87 ± 0.01 d	350.38 ± 0.14 e
GR-1-BBGK-19,568	78.15 ± 0.02 e	88.41 ± 0.46 b	1.91 ± 0.02 b	500.22 ± 0.15 a
GR-1-BBGK-19,674	90.88 ± 0.02 c	95.31 ± 0.14 a	2.46 ± 0.02 a	398.32 ± 0.58 c
GR-1-BBGK-19,640	83.88 ± 0.03 d	95.37 ± 0.80 a	1.85 ± 0.03 b	390.30 ± 0.24 d
GR-1-BBGK-19,635	97.03 ± 0.30 b	95.71 ± 0.36 a	2.09 ± 0.30 ab	344.34 ± 0.55 f
GR-1-BBGK-19,504	215.46 ± 0.00 a	95.71 ± 0.00 a	2.00 ± 0.08 b	71.85 ± 0.28 g
Average	101.71 ± 48.65	94.53 ± 2.59	1.80 ± 0.50	354.50 ± 128.21

Values represent mean values ± Standard Deviation (S.D.) of samples analyzed in triplicate (*n* = 3); Values with different letters in the same column are statistically significant (Tukey post-hoc test, *p* < 0.05).

**Table 2:** Results of preliminary propagation trials on different Greek native genotypes of *R. canina* utilizing the initial material collected directly from wild-growing populations. The table summarizes the most successful treatments used in terms of rooting frequencies. Original data are included in Table S1.

Population Sample (ACN)	Hormone Treatment (ppm IBA)	Mother Plant Development Stage **	Season of Year	Cutting Type	Rooting (%)
GR-1-BBGK-19,191	10,000	Dormancy	Winter	Hardwood cuttings	44.05
GR-1-BBGK-19,191	2500 *	Early growth (bud break)	Spring	Softwood cuttings	75.70
GR-1-BBGK-19,193	4000	Dormancy	Winter	Hardwood cuttings	10.71
GR-1-BBGK-19,193	2500 *	Early growth	Spring	Softwood cuttings	77.80
GR-1-BBGK-19,674	4000	Late growth	Autumn	Semi-hardwood cuttings	25.00
GR-1-BBGK-19,568	4000	Advanced growth	Summer	Softwood cuttings	1.30
GR-1-BBGK-19,579	2000	Advanced growth	Summer	Softwood cuttings	12.22
GR-1-BBGK-19,635	4000	Advanced growth	Summer	Softwood cuttings	28.00

* Hormone treatment applied through powdering, ** Early, advanced and late growth refer to the annual vegetative growth cycle.

**Table 3:** Multifaceted assessment of Greek native *Rosa canina* genotypes based on molecular authentication achieved, (Figure 1), success of preliminary propagation trials (see Table 2 for details), and relative phytochemical interest in terms of comparative vitamin C content (very high: >400 mg/100 g; high: >340–400; Low: <340) and total phenolic content (very high: >90 of gallic acid/100 g; high: 80–90; low: <80 mg).

IPEN Accession Number	DNA Barcoding	Success of Propagation Trials	Comparative Vitamin C Content	Comparative Total Phenolic Content
GR-1-BBGK-19,74	Effective	-	-	-
GR-1-BBGK-19,504	Effective	-	Low	Very high
GR-1-BBGK-19,560	Effective	-	-	-
GR-1-BBGK-19,568	Effective	Low	Very high	Low
GR-1-BBGK-19,635	-	Low	High	Very high
GR-1-BBGK-19,640	-	-	High	High
GR-1-BBGK-19,674	-	Low	High	Very high
GR-1-BBGK-03,2229	Effective	High	High	Low
GR-1-BBGK-19,186	Effective	-	-	-
GR-1-BBGK-19,191	Effective	High	Very high	High
GR-1-BBGK-19,193	Effective	High	-	-
GR-1-BBGK-19,579	Effective	Low	-	-

**Table 4:** Rooting attributes of the prioritized *Rosa canina* genotype GR-1-BBGK-19,191 expressed as rooting percentage (%) and mean values (±SEM, *p* < 0.05) of root number and average root length (mm) of rooted cuttings for each hormone treatment (ppm IBA) and substrate type (rooting experiment of summer 2019). All cuttings were soft-wood, leafy sections of the first growth year. The two substrate type ratios shown refer to perlite/peat (*v/v*) under mist conditions. Values within each column that do not share the same letter are significantly different (Tukey HSD, *p* < 0.05). Original data are included in Table S2.

Substrate Type	Cutting Type	Hormone Treatment (ppm IBA)	Rooting (%)	Root Number	Root Length (mm)
3:1	Apical Cuttings	Control	0	0	0
		1000	12.5	4.00 (±0.00) *	28.50 (±0.00)
		2000	12.5	7.00 (±0.00)	43.57 (±0.00)
		4000	0	0	0
		6000	0	0	0
	Sub-apical Cuttings	Control	0	0	0
		1000	0	0	0
		2000	37.5 ^†^	5.00 (±1.52) a	33.57 (±9.93) a
		4000	25.0	5.00 (±2.00) a	23.07(±9.07) a
		6000	0	0	0
1:1	Apical Cuttings	Control	0	0	0
		1000	0	0	0
		2000	0	0	0
		4000	37.5 ^†^	6.33 (±2.60) a	51.79 (±9.31) a
		6000	0	0	0
	Sub-apical Cuttings	Control	0	0	0
		1000	12.5	1.00 (±0.00)	13.00 (±0.00)
		2000	25.0	3.50 (±2.50) a	40.91 (±13.08) a
		4000	0	0	0
		6000	12.5	22.00 (±0.00)	18.59 (±0.00)

The ^†^ symbol denotes the highest rooting frequency following pairwise comparisons of the observed rooting frequencies via Pearson X^2^ tests. * In cases where only one replicate cutting managed to root, the standard error of the means for root number and length is 0.0 because they stem from a single value, as such those means are not included in the post-hoc test.

**Table 5:** Rooting attributes of the prioritized *Rosa canina* genotype GR-1-BBGK-03,2229 in experiments of 2019 (A) and 2020 (B) expressed as rooting percentage (%) and mean values of root number (±SEM, *p* < 0.05) and average root length (mm) of rooted cuttings for each hormone treatment (ppm IBA) and substrate type. All cuttings were soft-wood, leafy sections of the first growth year. The two substrate type ratios shown refer to perlite/peat (*v/v*) under mist conditions. Values within each column that do not share the same letter are significantly different (Tukey HSD, *p* < 0.05, lowercase letters for 2019 and capital letters for 2020). Original data are included in Table S3.

Substrate Type	Treatment	Rooting (%)	Root Number	Root Length (mm)
A				
3:1	Control	6.25	1.00 (±0.00) **	30.00 (±00.00)
	1000	0	0	0
	2000	12.25	5.50 (±1.50) a	26.17 (±13.67) a
	4000	6.25	6.00 (±0.00)	21.33 (±00.00)
	2500 *	25.00	3.00 (±0.91) a	19.55 (±5.15) a
1:1	Control	6.25	3.00 (±0.00)	56.00 (±00.00)
	1000	12.25	6.00 (±1.00) a	43.14 (±12.85) a
	2000	12.25	7.00 (±4.00) a	80.08 (±27.25) a
	4000	25	8.25 (±1.18) a	52.54 (±8.88) a
	2500 *	12.25	7.00 (±3.00) a	42.72 (±9.47) a
B				
3:1	Control	0	0	0
	2000	16.7	1.00 (±0.16) A	6.00 (±4.83) A
	4000	66.7 ^†^	3.00 (±1.71) A	14.75 (±6.65) A

* Hormone treatment applied through powdering. The ^†^ symbol denotes the highest rooting frequency following pairwise comparisons of the observed rooting frequencies via Pearson X^2^ tests. ** In cases where only one replicate cutting managed to root, the standard error of the means for root number and length is 0.0 because they stem from a single value, as such those means are not included in the post-hoc test.

**Table 6:** Rooting attributes of the Greek native *Rosa canina* genotype GR-1-BBGK-19,674 of summer 2020 trials expressed as rooting percentage (%) and mean values (±SEM, *p* < 0.05) of root number and average root length (mm) of rooted cuttings for each fertilization status and hormone treatment (ppm IBA) of mother plants. All cuttings were soft-wood, leafy sections of first growth year. The substrate type used was 3:1 perlite/peat (*v/v*) under mist conditions. Values within each column that do not share the same letter are significantly different (Tukey HSD, *p* < 0.05). Original data are included in Table S4.

Mother Plant Fertilization Status	Hormone Treatment (ppm IBA)	Rooting (%)	Root Number	Root Length (mm)
No fertilization	Control	33.3	2.50 (±0.50) a	20.41 (±2.91) a
	2000	16.7	2.00 (±0.00) *	5.00 (±0.00)
Conventional	Control	33.3	2.00 (±1.00) a	67.50 (±42.52) a
	2000	50.0 ^†^	3.33 (±0.33) a	87.77 (±11.39) a
Organic	Control	16.7	3.00 (±0.00)	86.67 (±0.00)
	2000	16.7	2.00 (±0.00)	92.50 (±0.00)

The ^†^ symbol denotes the highest rooting frequency following pairwise comparisons of the observed rooting frequencies via Pearson X^2^ tests. * In cases where only one replicate cutting managed to root, the standard error of the means for root number and length is 0.0 because they stem from a single value, as such those means are not included in the post-hoc test.

**Table 7:** Selected *Rosa canina* genotypes sampled from various mountainous habitats of northern Greece assigned with different IPEN (International Plant Exchange Network) accession numbers.

IPEN Accession Number	Greek Prefecture	Area	Altitude (m)	Sampling
GR-1-BBGK-19,74	Central Macedonia	Mt Voras	862	LS
GR-1-BBGK-19,504	Central Macedonia	Kastaneri	780	RR, LS
GR-1-BBGK-19,560	Central Macedonia	Mt Vermio	1615	LS
GR-1-BBGK-19,568	Central Macedonia	Mt Tzena	1086	SWSC, RR, LS
GR-1-BBGK-19,635	Central Macedonia	Mt Kroussia	650	SWSC, RR
GR-1-BBGK-19,640	Central Macedonia	Mt Kroussia	700	RR
GR-1-BBGK-19,674	Western Macedonia	Ziaka	900	SWSC, RR
GR-1-BBGK-03,2229	Epirus	Ioannina	650	SWSC, RR, LS
GR-1-BBGK-19,186	Epirus	Mt Lakmos	1370	LS
GR-1-BBGK-19,191	Epirus	Anogeia	1081	SWSC, RR, LS
GR-1-BBGK-19,193	Epirus	Mt Xirovouni	1070	SWSC, LS
GR-1-BBGK-19,579	Epirus	Pades	1180	SWSC, LS

SWSC: Soft-wood stem cuttings for propagation; RR: Ripe rosehips for phytochemical analysis; LS: Leaf samples for DNA analysis.

## Data Availability

All data supporting the results of this study are included in the manuscript and datasets are available upon request.

## Supplementary Materials

The following are available online at https://www.mdpi.com/article/10.3390/plants10122634/s1, Table S1: Raw data used to create Table 4 regarding the number of roots and root length (mm) during the 2019 propagation experiment of the prioritized *Rosa canina* genotype GR-1-BBGK-19,191 for each substrate type, hormone treatment (ppm IBA, in a *w/v* solution dissolved in 50% ethanol) and cutting type, Table S2: Raw data used to create Table 5 regarding the number of roots and root length (mm) of the prioritized *Rosa canina* genotype GR-1-BBGK-03,2229 during propagation experiments of 2019 (A) and 2020 (B) for each hormone treatment (ppm IBA in a *w/v* solution dissolved in 50% ethanol), Table S3: Raw data used to create Table 6 regarding number of roots and root length (mm) of the Greek native *Rosa canina* genotype GR-1-BBGK-19,674 during the 2020 propagation experiment per mother plant fertilization status and hormone treatment (ppm IBA in a *w/v* solution dissolved in 50% ethanol), Table S4: Raw data used to create Table 2 regarding the observed rooting frequencies during the preliminary propagation trials on different Greek native genotypes of *Rosa canina* (initial material collected directly from wild growing populations). The Table S4 summarizes the most successful treatments in terms of total number of replicate cuttings set for rooting depending on the availability of the collected material along with details on mother plant developmental stage, season and cutting type, presenting the frequencies of rooted cuttings out of the total number of replicate cuttings set in each treatment.
